# Integration of National Health Insurance claims data and animal models reveals fexofenadine as a promising repurposed drug for Parkinson’s disease

**DOI:** 10.1186/s12974-024-03041-7

**Published:** 2024-02-21

**Authors:** Jae-Bong Kim, Yujeong Kim, Soo-Jeong Kim, Tae‑Young Ha, Dong-Kyu Kim, Dong Won Kim, Minyoung So, Seung Ho Kim, Hyun Goo Woo, Dukyong Yoon, Sang Myun Park

**Affiliations:** 1 Department of Pharmacology, Ajou University School of Medicine, 164, Worldcup-Ro, Yeongtong-Gu, Suwon, 16499 Korea; 2 Center for Convergence Research of Neurological Disorders, Ajou University School of Medicine, Suwon, Korea; 3 Neuroscience Graduate Program, Department of Biomedical Sciences, Ajou University School of Medicine, Suwon, Korea; 4 Department of Biomedical Systems Informatics, Yonsei University College of Medicine, Seoul, Korea; 5 Standigm Inc., Seoul, Korea; 6 Department of Physiology, Ajou University School of Medicine, Suwon, Korea; 7 Present Address: Neuroscience Research Institute, Gachon University, Incheon, Korea

**Keywords:** Parkinson’s disease, α-Synuclein, Drug repositioning, Antihistamine, Fexofenadine

## Abstract

**Background:**

Parkinson’s disease (PD) is a common and costly progressive neurodegenerative disease of unclear etiology. A disease-modifying approach that can directly stop or slow its progression remains a major unmet need in the treatment of PD. A clinical pharmacology-based drug repositioning strategy is a useful approach for identifying new drugs for PD.

**Methods:**

We analyzed claims data obtained from the National Health Insurance Service (NHIS), which covers a significant portion of the South Korean population, to investigate the association between antihistamines, a class of drugs commonly used to treat allergic symptoms by blocking H1 receptor, and PD in a real-world setting. Additionally, we validated this model using various animal models of PD such as the 6-hydroxydopmaine (6-OHDA), α-synuclein preformed fibrils (PFF) injection, and *Caenorhabditis elegans* (*C. elegans*) models. Finally, whole transcriptome data and Ingenuity Pathway Analysis (IPA) were used to elucidate drug mechanism pathways.

**Results:**

We identified fexofenadine as the most promising candidate using National Health Insurance claims data in the real world. In several animal models, including the 6-OHDA, PFF injection, and *C. elegans* models, fexofenadine ameliorated PD-related pathologies. RNA-seq analysis and the subsequent experiments suggested that fexofenadine is effective in PD via inhibition of peripheral immune cell infiltration into the brain.

**Conclusion:**

Fexofenadine shows promise for the treatment of PD, identified through clinical data and validated in diverse animal models. This combined clinical and preclinical approach offers valuable insights for developing novel PD therapeutics.

**Supplementary Information:**

The online version contains supplementary material available at 10.1186/s12974-024-03041-7.

## Background

Parkinson’s disease (PD) is a common costly and devastating progressive neurodegenerative disease. The main pathological features of PD are the progressive loss of dopaminergic neurons located in the substantia nigra pars compacta and widespread intracellular proteinaceous inclusions called Lewy bodies (LBs) and Lewy neurites (LNs) in the remaining neurons. Although the etiology of PD is not fully understood, α-synuclein (α-syn), as a major component of LBs and LNs, and a gene whose mutations have been identified in familial PD, is thought to play an important role in the pathogenesis of PD [1]. The notion that prion-like propagation of α-syn induces PD progression also supports the importance of α-syn [2, 3]. In addition, oxidative stress, mitochondrial damage, and defects in the protein quality control system play roles in the pathogenesis of PD [1, 4]. Recent Mendelian randomization study and multi-omics research have underscored the significance role of neuroinflammation in PD [5, 6].

With increasing life expectancy, the prevalence of this disease is rapidly increasing. Although dopamine supplements, such as levodopa, and dopamine agonists have been used as symptomatic treatments, a disease-modifying approach that can directly stop or slow the progression of PD remains a major unmet need in its treatment [1].

There are many strategies for developing disease-modifying drugs for PD. Among these, the clinical pharmacology-based drug repositioning strategy is a very useful approach for identifying new drugs against PD that are efficient, safe, and inexpensive [7]. In particular, real-world data such as electronic health records, medical imaging, and genomic data have been increasingly used to support the drug development process because they better represent the actual pattern of drug treatment and outcomes in the real world [8, 9]. National Health Insurance claims data can also reveal patterns of drug use and outcomes in real-world patient populations, which can help identify potential new uses of drugs [10, 11].

Previous studies suggested that histamine is involved in the pathogenesis of PD. PD patients have significantly higher blood histamine levels than normal controls [12]. In postmortem brain samples from patients with PD, histamine concentrations are significantly elevated compared to age-matched controls [13]. Histamine induces microglia activation and dopaminergic neuronal cell death via H1 receptor [14]. Endogenous histamine also accelerates the degeneration of dopaminergic neurons via its H1 receptor in 6-hydroxydopmaine (6-OHDA) lesioned rats [15], suggesting that the histamine system may be a target against PD.

In the present study, we analyzed claims data obtained from the National Health Insurance Service (NHIS), which covers a significant portion of the South Korean population, to investigate the association between antihistamines, a class of drugs commonly used to treat allergic symptoms by blocking H1 receptor, and PD in a real-world setting. The selected antihistamine was administered to mice, and its effects and mechanisms of action were studied using behavioral assessments and RNA-seq analysis.

## Materials and methods

### Investigation of the association between antihistamines and PD in real-world settings

Our study focused on patients diagnosed with PD after the age of 40 years between 2002 and 2020. We included patients who were prescribed either PD drugs (amantadine, benserazide, carbidopa, entacapone, levodopa, pramipexole, rasagiline, ropinirole, or selegiline) or antihistamines (azelastine, bepotastine, cetirizine, chlorpheniramine, clemastine, desloratadine, dimenhydrinate, ebastine, fexofenadine, hydroxyzine, ketotifen, levocetirizine, loratadine, mizolastine, or olopatadine). Patients with missing information in their prescription records, such as the prescription period, daily dose, or single dose, were excluded. For the antihistamine-exposed group, we included patients who had continuous use of antihistamines for a duration exceeding one month (31 days). We also considered two prescriptions with an interval of less than one month (31 days) for continuous use of antihistamines. Furthermore, we included only individuals who had taken a single type of antihistamine. The antihistamine-unexposed group comprised patients who did not meet the exposure criteria. We examined the prescription records for both the antihistamine-exposed and unexposed groups over a 3-month period before and after the index date. The index date was defined as the date of the first antihistamine prescription for the exposed group. Then we calculated the time interval between the first PD diagnosis and the date of the first antihistamine prescription and its median time (in days). For the antihistamine-unexposed group, we added the median time to the first PD diagnosis date for each individual in the unexposed group and used it as an index date. This strategy ensures that the index date for the unexposed group matches the common timeframe seen in the exposed group. As a measure, we chose levodopa equivalent daily dose (LEDD) as our primary outcome because it has been reported that LEDD changes can be used as a disease progression marker [16], and LEDD was also reported as a measure in studies that evaluated either the clinical efficacy of second-line therapies or their impact on medication regimens [17]. The LEDD was calculated by multiplying the levodopa equivalent dose (LED) with the levodopa prescription period for each patient. The LED was determined by multiplying the conversion factor of each PD drug by the levodopa dose (https://www.parkinsonsmeasurement.org/toolBox/levodopaEquivalentDose.htm). We employed propensity score matching to mitigate the potential confounding factors. The nearest neighbor method was used to establish a 1:3 matching ratio between the exposed and unexposed groups, with a caliper value of 0.25 applied for the matching. The two groups were matched based on sex, age at PD diagnosis, levodopa prescription period, and LEDD values during the 3 months preceding the index date. The difference and increment ratio of the LEDD for the 3-month period before and after the index date were then calculated. The increment ratio was defined as follow formula:

$$\mathrm{Increment\, ratio}=100 \times \frac{1}{N}\sum_{i=1}^{N}\frac{{{\text{LEDD}}}_{{\text{after}},i}-{{\text{LEDD}}}_{{\text{before}}, i}}{{{\text{LEDD}}}_{{\text{before}},i}}.$$

In this formula, *N* represents the total number of subjects in the study. LEDD_after*,i*_ denotes the LEDD for the *i*th patient after the index date, and LEDD_before,*i*_ denotes the LEDD for the *i*th patient before the index date. This ratio, multiplied by 100, provides the percentage change in LEDD for each patient, and the average of these changes across all patients gives the overall increment ratio. Therefore, a small increment ratio indicates slower progression of PD. The increment ratios between the exposed and unexposed groups were compared using a two-tailed unpaired t-test. For this study, we used the Statistical Analysis System Enterprise Guide version 7.1 (SAS Institute Inc., Cary, NC, USA) and R Studio version 3.3.3 for data extraction and statistical analysis. MatchIt package 4.5.0 version was used for propensity score matching in R Studio.

### Antibodies and reagents

Primary antibodies against pSer129 α-syn (#015-25191) and IBA-1 (#019-19741) were purchased from Wako Pure Chemical Industries (Richmond, VA, USA). The primary antibody against glial fibrillary acidic protein (GFAP, #RA22101) was purchased from Neuromics (Edina, MN, USA). Primary antibodies against CD3 (#ab16669) and tyrosine hydroxylase (TH, #ab137721) were purchased from Abcam (Cambridge, UK). The primary antibody against F4/80 (#sc-377009) was purchased from Santa Cruz Biotechnology (Dallas, TX, USA). Primary antibody against NCR1 (#PA5-102,860) was purchased from Thermo Fisher Scientific (Waltham, MA, USA). Fexofenadine (#F9427) was purchased from Sigma Aldrich (St. Louis, MO, USA).

### Animals

WT C57BL/6J mice were purchased from Daehan Biolink (Eumseong, Korea). M83 transgenic (Tg) mice, which are A53T mutant α-syn protein expressing under the prion promoter, were purchased from The Jackson laboratory (Harbor, USA). Heterozygous M83 Tg mice were obtained by mating homozygous M83 Tg mice with C57BL/6J mice. All mice were housed in a specific-pathogen-free environment with a 12 h/12 h light–dark cycle and given free access to food and water.

### Stereotactic injection and drug treatment

For the 6-OHDA (#4381, Sigma Aldrich) injection model, 8- to 12-week-old C57BL/6J mice were anesthetized via intraperitoneal injection using 2,2,2-tribromoethanol (250 mg/kg) and injected into the unilateral striatum (Cpu) (coordinates: anterior–posterior, + 1.0 mm; medial–lateral, − 1.8 mm; dorsal–ventral, − 3.2 mm from the bregma) with sterile phosphate-buffered saline (PBS) or 2 mg/ml 6-OHDA dissolved in sterile saline containing 0.02% ascorbic acid. For the α-synuclein preformed fibrils (PFF) injection model, the injection protocol was as previously described [18]. Briefly, 8- to 12-week-old M83 Tg mice followed the protocol identical to the 6-OHDA injection model, receiving either sterile PBS or 10 μg PFF. After recovery from surgery, mice in the 6-OHDA injection model were fed with 200 μl vehicle (sterile water) or fexofenadine 20 mg/kg/day by oral gavage for 3 weeks, and mice in the PFF injection model were fed with vehicle or the same drugs for 4 weeks. Administering fexofenadine to mice at 20 mg/kg/day via oral gavage was determined on assuming a human prescription dose of 120 mg/day and calculating the animal equivalent dose [19] and several references that demonstrated effective outcome in mice [20–22]. The vehicle and drugs were dissolved in 0.5% hydroxypropyl methylcellulose and 0.1% Tween-80 in 200 μl sterile water.

### Mouse behavioral test

In the pole test, the mice were placed facing head-up on top of a wooden pole (50 cm in height and 1 cm in diameter), and the total time taken to reach the base of the pole was recorded. The maximum cutoff time to stop the test and record was 60 s. Before the main test, the mice were trained for two consecutive days. On the main test day, mice were subjected to three trials. In the cylinder test, each mouse was immediately placed in a 500-ml beaker and acclimatized for 2 min prior to testing. Video recording was started after acclimatization and continued until the number of left, right, or both forelimb wall contacts were countered until the total number of wall contact reached 20. The data are presented as frequency ratios of the left to both forelimb wall touches. The grip strength test was performed using a grip strength meter (Ugo Basile, Gemonio, VA, Italy). In the bridge test, mice were placed on the right side of a wooden beam (100 cm length, 1 cm width) at a height of 50 cm from the floor, and the total time taken to cross to the left side of the beam was recorded. The maximum cutoff time to stop the recording was 120 s. Mice were trained for three consecutive days.

### Immunohistochemistry and quantification

The mice were intraperitoneally anesthetized with urethane (200 mg/kg) and perfused with PBS and 4% paraformaldehyde (pH 7.4). The brains were collected, post-fixed overnight with 4% paraformaldehyde, and cryoprotected in 30% sucrose in PBS for 3–4 days. Fixed brains were frozen in OCT compounds and cut with a cryo microtome (Leica Microsystems, Wetzlar, Germany) at 35 μm thickness. Free-floating sections were washed three times for 10 min in PBS and treated with 3% hydrogen peroxide (#H1009, Sigma Aldrich) in PBS for 5 min to inactivate endogenous peroxidase. Sections were blocked with 1% bovine serum albumin and 0.2% Triton X-100 in PBS for 1 h. Primary antibodies were dissolved in blocking buffer and incubated for 24 h. After washing with PBS, sections were incubated with a biotinylated secondary antibody (Vector Laboratories, Burlingame, CA, USA) for 1 h at room temperature. Sections were then washed with PBS and incubated with avidin–biotin complex reagent (Vector Laboratories) for 1 h. Sections were stained using 0.05% 3′3-diaminobenzidine (Sigma-Aldrich) in 0.1 M phosphate buffer, pH 7.2 at room temperature, and mounted on slides. The sections were dehydrated with graded ethanol (70–100%), cleaned in xylene, mounted with a per-mount solution, and covered with a coverslip. All stained tissue images were obtained using an Axio Scan Z1 Slide Scanner (Carl Zeiss, Oberkochen, Germany). For quantification of pSer129 α-syn pathology, we counted numbers of pSer129 α-syn pathology using the ZenBlue 2.3 software (Carl Zeiss). We selected reference coronal section images, relative to bregma: 2.1 mm, 0.98 mm, 0.14 mm, − 1.58 mm and − 3.08 mm. Next, we calculated the average pSer129 α-syn pathology per animal, and then calculated average numbers per group. For measurement of IBA-1 density, we counted the numbers of IBA-1 positive cells at 20 × magnification using ZenBlue 2.3 software. Cell density was calculated by dividing the measured value by the area at 20 × magnification, providing the number of IBA-1 positive cell per 1 mm^2^. GFAP intensity was measured as described previously [18]. The quantification of TH-positive neurons was performed as previously described [23]. Briefly, substantia nigra (SN) sections were collected in a 1:6 series with a thickness of 35 μm, obtaining one well per mouse and subsequent analyzing 5–6 free-floating sections. Numbers represent the total number of TH-positive cells per mouse multiplied by six to calculate the population estimate.

### *Caenorhabditis elegans* model system

All Bimolecular Fluorescence Complementation (BiFC) Tg worms which were generated as previously described [24] and maintained on nematode growth medium (NGM) plates fed with *Escherichia coli* (*E. coli*) strain OP50 at 20 °C. Three representative BiFC Tg lines were used. To analyze the effects of fexofenadine on the aggregate transmission of Tg worms, L4-stage worms were transferred to NGM plates with 10 μM fexofenadine, and then all Tg worms treated with the drug were maintained at 20 °C. For imaging of the BiFC Tg worms, worms immobilized with 10 mM sodium azide (#S2002, Sigma-Aldrich) in M9 buffer (22 mM KH_2_PO_4_, 22 mM Na_2_HPO_4_, 85 mM NaCl, and 1 mM MgSO_4_) were placed on a microscope slide (#HSU-0101242, Marienfeld Laboratory Glassware, Lauda-Königshofen, Germany) and covered with a coverslip. All images were obtained using a Leica confocal laser scanning system (Leica Microsystems). To analyze the VENUS-BiFC fluorescence intensity, the integrated level of VENUS fluorescence and the area of the pharynx were measured. Subsequently, the intensity of VENUS fluorescence was defined as the relative fluorescence intensity per pharyngeal area using the Leica Las-X software (Leica Microsystems). The pharyngeal pumping of WT N2 and Tg worms was measured for 1 min at room temperature using a Leica Microscope and Imaging System (Leica Microsystems). The rate of the pumps was denoted as pumps per minute. Lifespan assays were performed as described previously [24]. The synchronized progeny from eggs were grown to reach L4-stage worms, and the worms were transferred to NGM plates, containing 50 μM 5-fluoro-2′-deoxyuridine (#F0503, Sigma-Aldrich) to prevent hatching of progeny, with 10 μM fexofenadine treatment. The number of live and dead worms was monitored and counted daily. Worms that ruptured, burrowed, or crawled off the plates were censored but were included in the lifespan analysis as censored animals. The mean lifespan was analyzed using an online application for survival analysis (OASIS 2; https://sbi.postech.ac.kr/oasis2/) [25].

### Bulk RNA-sequencing and data analysis

To extract total RNA from the cortex of the M83 mouse brain, 2- to 3-month-old hemizygous M83 mice were anesthetized with an intraperitoneal injection of urethane. Extracted ipsilateral cortex tissue was immediately frozen by liquid nitrogen and stored at -80℃ until use. The tissues were homogenized using a cordless homogenizer (Kimble, USA) and individually extracted using a Qiagen RNeasy Mini Kit (Qiagen, Hilden, Germany). Total RNA was eluted in a 40 μl elution buffer. RNA quality and concentration were measured using the Nanodrop2000 spectrometer. The RNA libraries were sequenced on an Illumina NovaSeq 6000 system 150PE for 150 bp paired-end reads with coverage > 52 million reads per sample by Teragen Etex Bio (Suwon, Korea). The data parsing for measuring RNA abundance was performed using the R package “SEQprocess”. RNA abundance was calculated using FASTQC (version 0.11.9), Trim Galore (version 0.6.6), and Cufflinks (version 2.2.1). The reads were aligned to the *Mus musculus* genome reference consortium build (GRCm38) using STAR (version 2.7.3a). Transcriptome data were analyzed by log2 transformation, quantile normalization, and the gene centering method. Differentially expressed genes (DEGs) between the PBS and PFF models was analyzed using the Student’s t-test. Functional gene clusters were determined by evaluating functional enrichment using Gene Ontology (GO). Data from GO: BP, KEGG (http://www.genome.jp/kegg), and Reactome (http://www.reactome.org) analyses using the gProfileR (version 0.7.0) package in R software (version 4.2.1). Significant gene clusters with a p-value below 0.05 were selected for further analysis. Statistical analysis of RNA-seq data was carried out using R software.

### Ingenuity pathway analysis

To analyze pathways and networks from the RNA-seq data, we used the Ingenuity Pathway Analysis (IPA) bioinformatics software with the Qiagen Knowledge Base (https://www.qiagenbioinformatics.com/products/ingenuity-pathway-analysis). We uploaded the DEGs data into IPA. Canonical pathway analysis was performed to identify the significantly enriched biological pathways among the DEGs based on the ratio, *p*-value, and *Z*-score. The ratio was calculated as the number of DEGs in the dataset divided by the total number of genes in the canonical pathway database. The *Z*-score represents the deviation from the expected activation state of a canonical pathway, based on the expression of the genes in the data set. The *p*-value for canonical pathway analysis was calculated using Fisher's exact test. Additionally, an Upstream Regulator Analysis was performed to predict upstream regulators that could potentially be responsible for the observed gene expression changes based on the Z-score.

### Real-time PCR (RT-PCR)

Reverse transcription was performed using 1 μg RNA and a qMAX cDNA Synthesis kit (#PR2100, Accuris, Edison, NJ USA). RT-PCR was performed with TB Green Premix Ex Taq (#RR420A, Takara Bio, Kusatsu, Shiga, Japan) using a Thermal Cycler Dice Real-Time System Single machine (Takara Bio), with CD3, F4/80, NCR1, or GAPDH as internal controls. Gene expression was analyzed using the 2-ΔΔCt method. A complete list of primer sequences is provided in Additional file 1: Table S1.

### Statistical analysis

All values are expressed as mean ± SEM, unless otherwise noted. Statistical significance was evaluated using unpaired *t*-test or one-way or two-way ANOVA with Tukey’s multiple comparison test, unless otherwise noted (GraphPad Prism software, San Diego, CA, USA).

## Results

### NHIS analysis predicts fexofenadine as a candidate drug for slowing the progression of PD

**Fig. 1:**
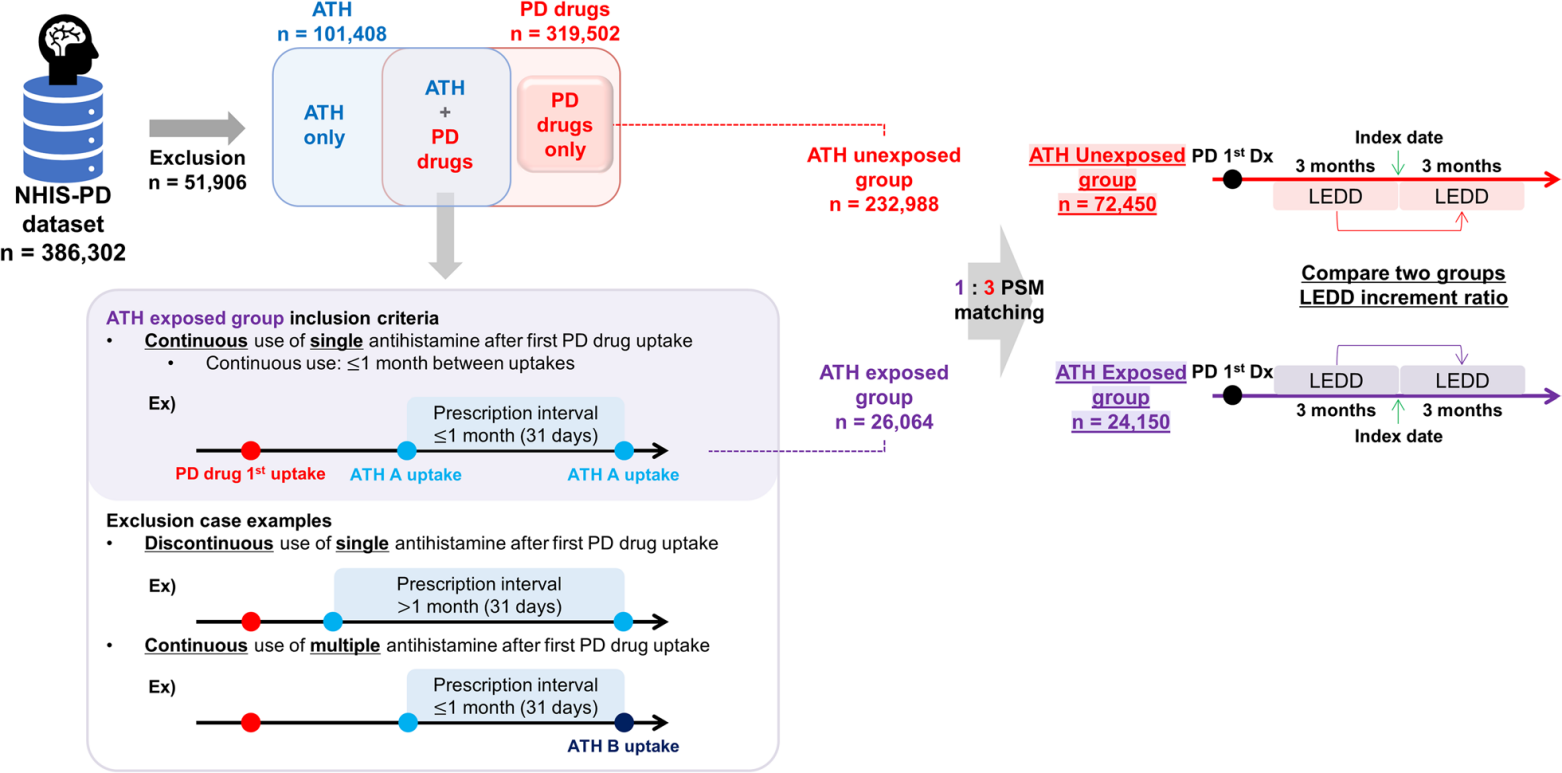
Overall research flow to select a drug candidate. Dataset provided by NHIS was divided into antihistamine-exposed and antihistamine-unexposed groups according to their prescription records. Each group’s LEDD before and after the index date was compared. *NHIS-PD* National Health Insurance System-Parkinson’s Disease, *PD* Parkinson’s disease, *ATH* antihistamines, *PSM* propensity score matching, *Dx* diagnosis, *LEDD* levodopa equivalent daily dose, *n* number of patients

In our analysis of the NHIS claims database in Korea, we included a total of 386,302 patients. Among them, 51,906 were excluded because they have at least one missing prescription record variables; the prescription period, daily dose, or single dose. 319,502 were prescribed PD drugs, 101,408 were prescribed antihistamines, and 86,514 received both types of medications. A subset of 26,064 patients was identified who continuously received only a single type of antihistamine. Following the 1:3 propensity score matching, the antihistamine-exposed group was adjusted to include 24,150 patients, while the antihistamine-unexposed group included 72,450 patients. The overall research flow is illustrated in Fig. 1. We first determined the median time (in days) from PD diagnosis to the index date in the antihistamine-exposed group. The median time of overall antihistamine was 752 days. For each antihistamine, their median time is listed in Additional file 1: Table S2. This value was then applied to each individual in the unexposed group to establish their respective index dates, ensuring a methodologically consistent comparison between the two groups. The primary outcome, changes in LEDD, was analyzed (Table 1). We observed that the increment ratio of LEDD in the overall antihistamine-exposed group was lower than that in the unexposed group. However, the difference between these groups was not statistically significant. Upon further analysis, we again found no significant differences by dividing the antihistamine-exposed group into first- and second-generation antihistamines. However, when we analyzed individual antihistamine drugs, notable findings emerged. Specifically, chlorpheniramine, fexofenadine, and levocetirizine showed significantly lower increment ratios of LEDD in the exposed group compared to the matched unexposed group, with p-values less than 0.05. Fexofenadine demonstrated the most substantial effect in our study. The increase in LEDD before and after the index date showed a marked difference between the groups: it was 13.537 (changing from 253.307 to 266.844) in the fexofenadine-unexposed group, compared to only 5.584 (from 253.276 to 258.860) in the exposed group. Notably, the increment ratio—an average value derived from individual patient ratios, where each ratio is the increment of LEDD relative to the baseline LEDD for that patient—was significantly lower for fexofenadine. This finding indicates an inhibitory effect, with the average ratio being 6.997 in the exposed group versus 9.399 in the unexposed group (*p* = 0.045). These results suggest that fexofenadine may play a role in slowing PD progression, warranting further investigation.

**Table 1:** The association between antihistamines and LEDD

Antihistamine	Exposed case	Antihistamine-unexposed group				Antihistamine-exposed group				
		LEDD	LEDD	Difference	Ratio	LEDD	LEDD	Difference	Ratio	*p*-value
		Before index	After index			Before index	After index			
Overall	24,150	258.143	263.982	5.839	5.607	263.127	267.32	4.193	6.208	0.17
1st generation	13,391	257.533	263.655	6.122	5.63	272.614	276.255	3.641	4.919	0.369
Chlorpheniramine	4832	256.555	260.456	3.901	4.575	289.544	290.461	0.916	3.35	0.011^*^
Clemastine	28	245.797	252.404	6.607	5.828	260.051	254.51	-5.54	0.833	0.378
Dimenhydrinate	6481	256.032	263.127	7.095	6.042	251.093	257.03	5.937	6.821	0.127
Hydroxyzine	2050	256.758	261.798	5.04	3.935	271.368	271.564	0.196	2.748	0.051
2nd generation	10,759	257.571	263.582	6.011	5.586	253.639	258.385	4.745	7.497	0.09
Azelastine	1098	258.29	264.081	5.791	5.807	280.206	284.477	4.271	4.977	0.681
Bepotastine	2086	256.223	263.149	6.926	6.328	252.281	256.457	4.176	5.229	0.243
Cetirizine	1685	261.267	267.499	6.232	5.422	268.424	273.625	5.201	6.118	0.492
Desloratadine	65	262.288	272.612	10.323	6.893	247.033	238.168	-8.865	3.057	0.341
Ebastine	599	260.882	267.265	6.383	6.278	260.019	272.092	12.073	9.224	0.088
Fexofenadine	1356	253.307	266.844	13.537	9.399	253.276	258.86	5.584	6.997	0.045^*^
Ketotifen	147	258.036	259.198	1.163	4.706	264.295	276.573	12.279	9.575	0.162
Levocetirizine	2384	257.333	269.701	12.373	8.309	258.232	263.3	5.068	6.35	0.046^*^
Loratadine	374	266.108	273.866	-7.758	5.128	261.734	256.611	-5.124	2.15	0.087
Olopatadine	965	259.044	268.828	9.784	8.341	209.294	235.14	25.846	27.218	< 0.001^***^

*p < 0.05, ***p < 0.001

### Fexofenadine protects dopaminergic neurons in the 6-OHDA-induced animal model of PD

**Fig. 2:**
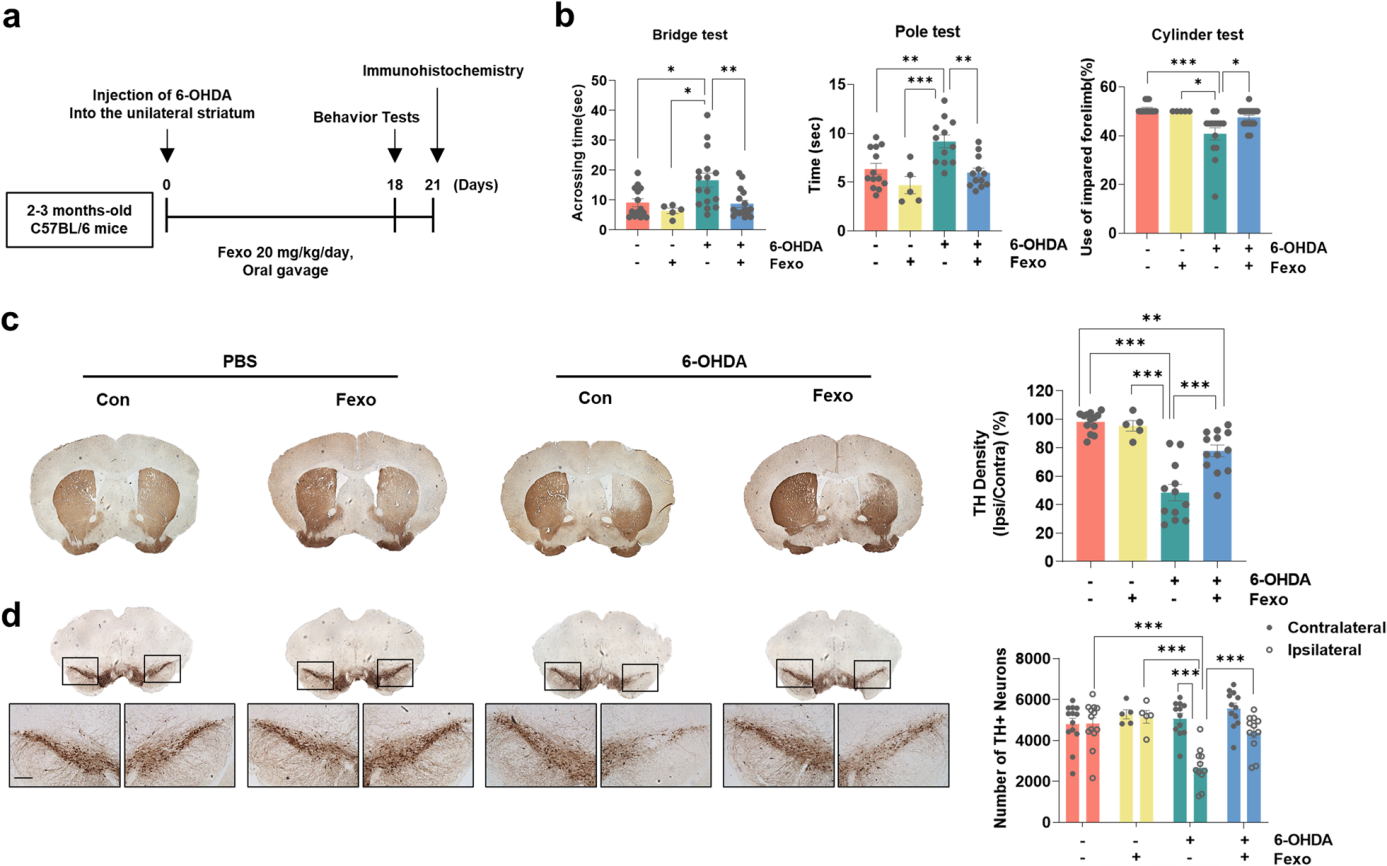
Fexofenadine ameliorates dopaminergic neuronal cell death in 6-OHDA mouse model. **a** Schematic diagram of in vivo 6-OHDA experiment. **b** Behavioral results of mice in the bridge, pole and cylinder tests at 3–4 months after PBS, PBS + Fexofenadine (Fexo), 6-OHDA, or 6-OHDA + Fexo groups. **c** IHC of TH in striatum (Cpu) and analysis of striatal TH density in PBS, PBS + Fexo, 6-OHDA, or 6-OHDA + Fexo. **d** IHC of TH in substantia nigra (SN) and analysis of stereology counts of TH-positive neurons in PBS (*n* = 13), PBS + Fexo (*n* = 5), 6-OHDA (*n* = 12), or 6-OHDA + Fexo (*n* = 12) groups. Scale bar indicates 200 μm. ***p < 0.001, **p < 0.01, *p < 0.05, one-way ANOVA with Tukey’s multiple comparison test

**Fig. 3:**
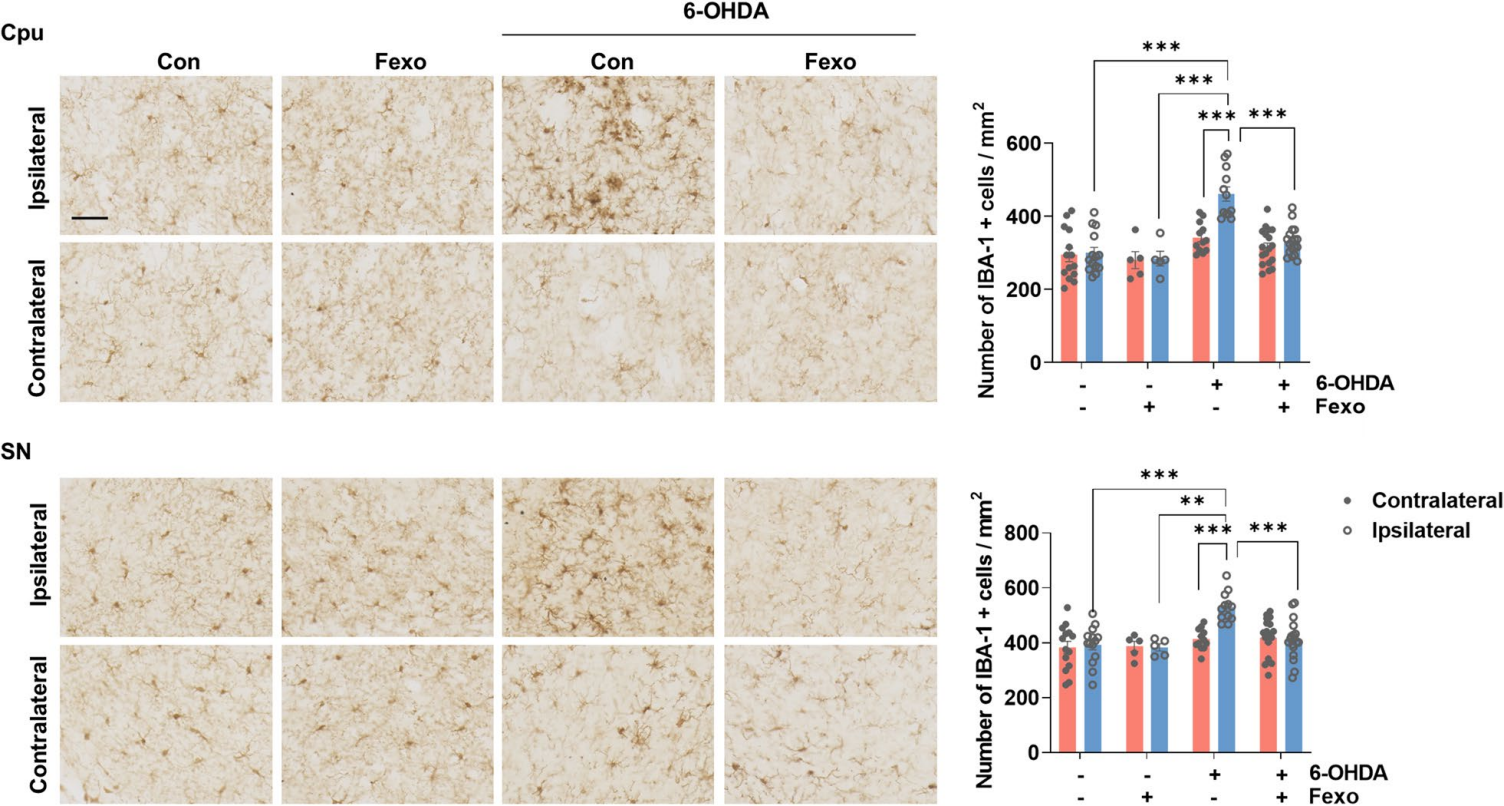
Fexofenadine ameliorates microgliosis in 6-OHDA mouse model. IHC of IBA-1 in Cpu and SN in PBS, PBS + Fexo, 6-OHDA, or 6-OHDA + Fexo. Values are derived from the analyzed density of IBA-1 positive cell density/mm^2^ in Cpu and SN in PBS (*n* = 14), PBS + Fexo (*n* = 5), 6-OHDA (*n* = 12), or 6-OHDA + Fexo groups (*n* = 18). Scale bar indicates 50 μm. ***p < 0.001, **p < 0.01, two-way ANOVA with Tukey’s multiple comparison test

To validate the potential utility of fexofenadine in the treatment of PD, we conducted experiments using a 6-OHDA-injected mouse model (Fig. 2a). Fourteen days after injecting 6-OHDA into the unilateral striatum, we conducted several motor behavior tests, including the bridge, pole, and cylinder tests. These tests are commonly used to evaluate the balance, forelimb asymmetry, and bradykinesia, respectively. The injection of 6-OHDA resulted in impaired motor behavior, whereas fexofenadine improved in the motor deficits induced by the 6-OHDA injection (Fig. 2b). Furthermore, the injection of 6-OHDA caused a significant decrease in the TH intensity in the Cpu and a reduction in the number of TH-positive cells in the SN compared to animals treated with PBS. This observation correlates with the results of the motor behavior tests. However, fexofenadine treatment significantly attenuated the loss of TH staining (Fig. 2c, d), indicating that fexofenadine administration effectively protected dopaminergic neurons from the toxicity induced by 6-OHDA injection. To visualize microglial reactivity, tissue sections from the dorsal Cpu and SN were stained with an anti-IBA-1 antibody. We observed a significant increase in microglial reactivity in 6-OHDA lesioned mice compared to the vehicle-treated group in both areas. In contrast, treatment with fexofenadine reduced microglial activation induced by 6-OHDA injection (Fig. 3), suggesting that fexofenadine administration diminished microgliosis triggered by 6-OHDA.

### Fexofenadine ameliorates α-syn propagation in the PFF-injected mouse model

**Fig. 4:**
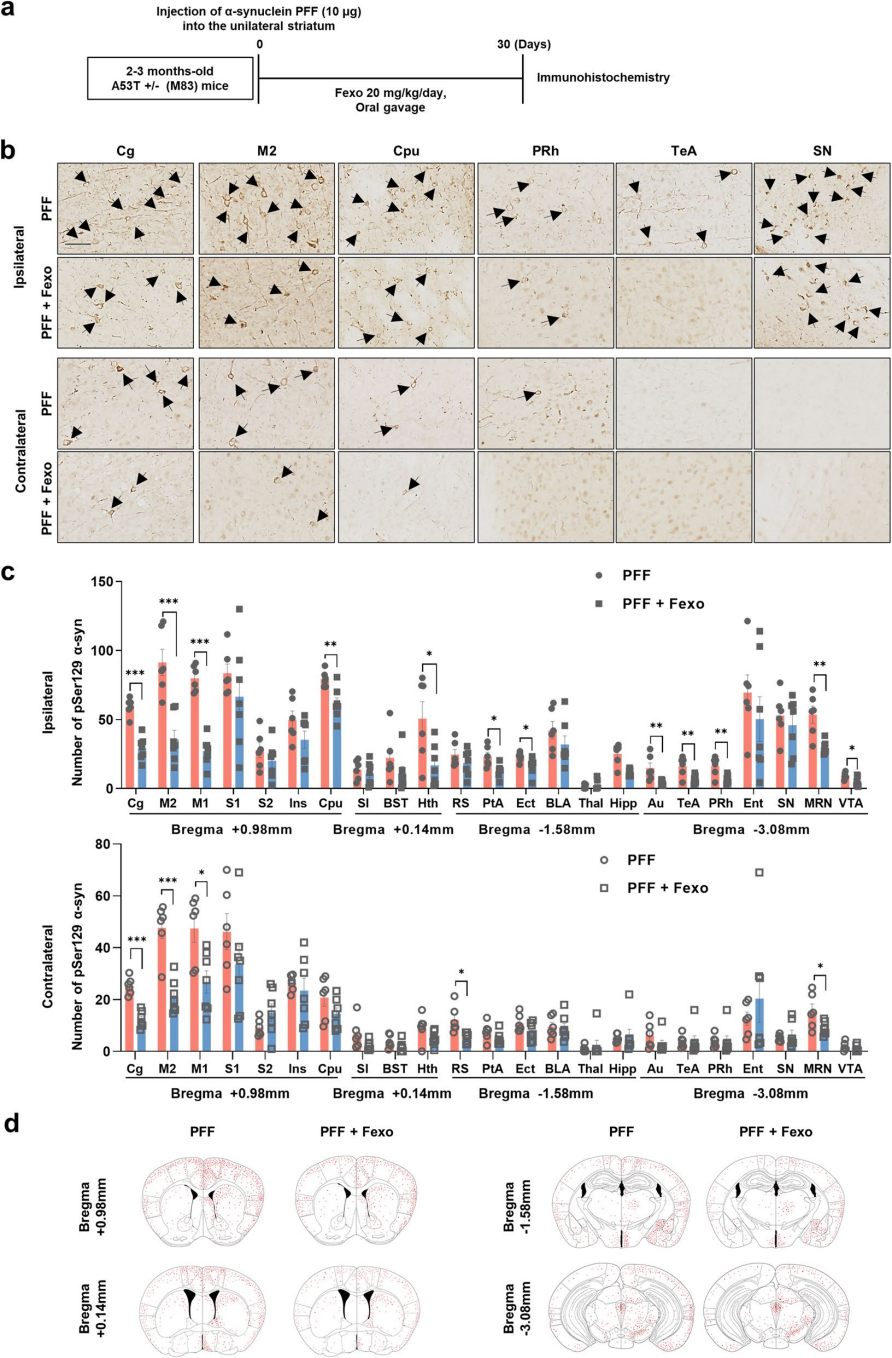
Fexofenadine inhibits the propagation of α-syn in PFF-injected mice. **a** Schematic diagram of in vivo PFF injection experiment. **b** IHC of pSer129 α-syn in PFF or PFF + Fexo. Arrows indicate positive inclusion bodies. Scale bar indicates 50 μm. **c** The number of pSer129 α-syn in ipsilateral and contralateral regions. Values are derived from the analysis of data collected from PFF (*n* = 6) or PFF + Fexo (*n* = 7) groups at 4 weeks post-injection. ***p < 0.001, **p < 0.01, *p < 0.05, one-way ANOVA with Tukey’s multiple comparison test. **d** The brain atlas indicates the distribution of pSer129 α-syn-positive inclusion bodies (red dots) in coronal sections from mice brains with PFF or PFF + Fexo groups at 4 weeks post-injection. See Additional file 1: Fig. S1 for abbreviations used

**Fig.5:**
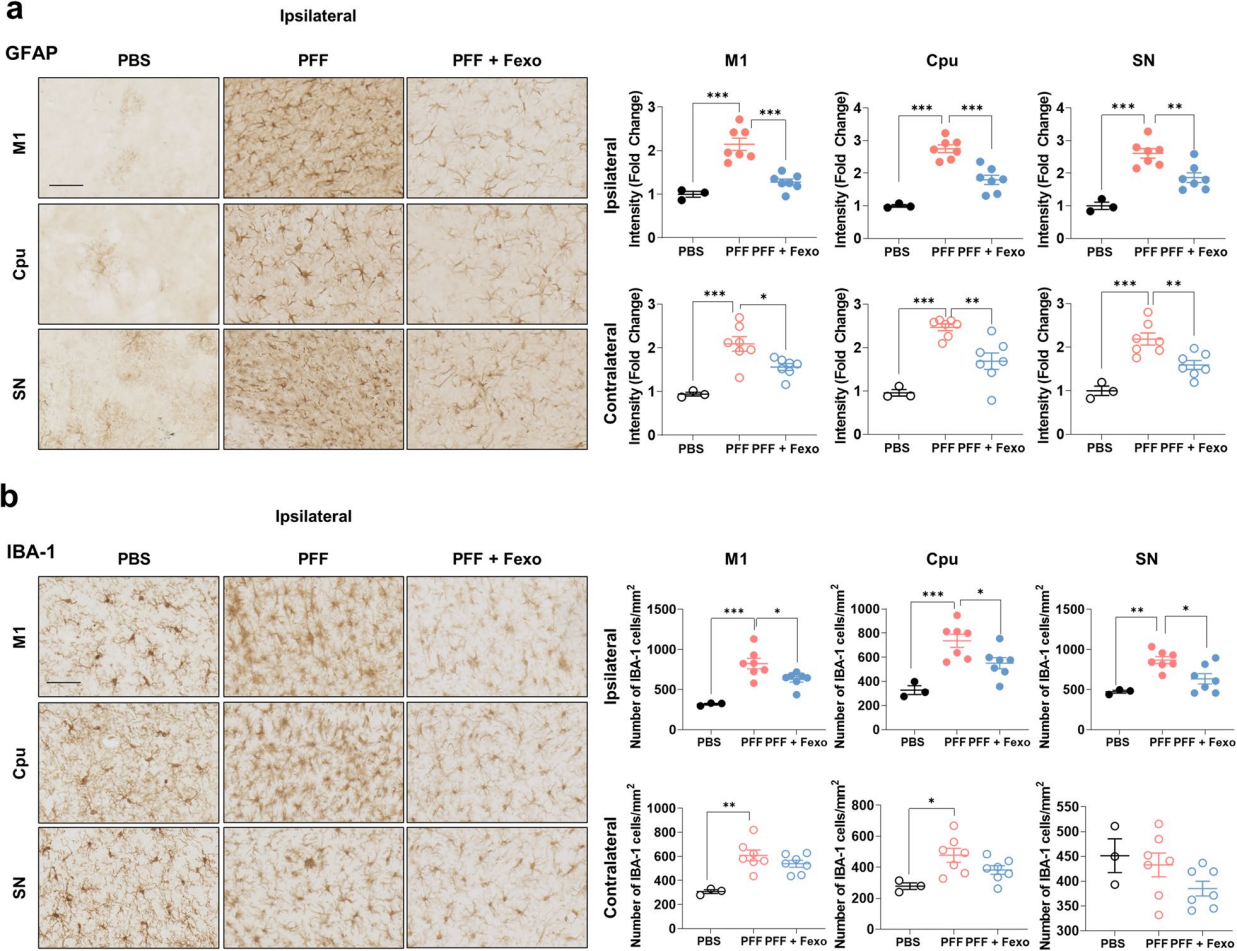
Fexofenadine mitigates micro/astro-gliosis in PFF-injected mice.** a** IHC of GFAP in ipsilateral M1, Cpu and SN regions of PFF or PFF + Fexo. Scale bar indicates 50 μm. Values are derived from the analysis of data collected from PBS (*n* = 3), PFF (*n* = 7), or PFF + Fexo (*n* = 7) groups at 4 weeks post-injection. **b** IHC of IBA-1 in ipsilateral M1, Cpu and SN regions of PFF or PFF + Fexo groups. Values are derived from the analysis of data collected from PBS (*n* = 3), PFF (*n* = 7) or PFF + Fexo (*n* = 7) groups at 4 weeks post-injection. Scale bar indicates 50 μm. ***p < 0.001, **p < 0.01, *p < 0.05, one-way ANOVA with Tukey’s multiple comparison test

To investigate the effect of fexofenadine on α-syn pathology, PFF were injected into the unilateral Cpu of 8-week-old A53T Tg mice (Fig. 4a). After a 30-day post-injection period, α-syn lesions were detected bilaterally in the brain using immunohistochemistry targeting pSer129 α-syn, a marker of pathological α-syn. This finding is consistent with that of a previous study [18]. To assess whether fexofenadine could reduce the propagation of α-syn in an in vivo animal model, fexofenadine was administered to 8-week-old A53T mice after the injection of PFF for one month. Notably, fexofenadine effectively reduced the propagation of α-syn lesions (Fig. 4b–d). In addition to evaluating the impact on α-syn pathology, we examined related neuroinflammation by analyzing astrocytes and microglia. Immunostaining for GFAP, a marker of astrocytic reactivity, revealed increased GFAP expression in various regions, including the cortex and the Cpu, following PFF injection. However, fexofenadine treatment reduced GFAP expression (Fig. 5a; Additional file 1: Fig. S2), suggesting the inhibition of astrogliosis. Similarly, the activation of microglia, as assessed by IBA-1 staining, was diminished by fexofenadine administration (Fig. 5b; Additional file 1: Fig. S3). This suggests that fexofenadine may have potential as a therapeutic approach in the context of α-syn-related disorders, offering benefits in terms of reducing α-syn aggregation and associated neuroinflammation.

### Fexofenadine ameliorates α-syn propagation in the *Caenorhabditis elegans* BiFC model

**Fig. 6:**
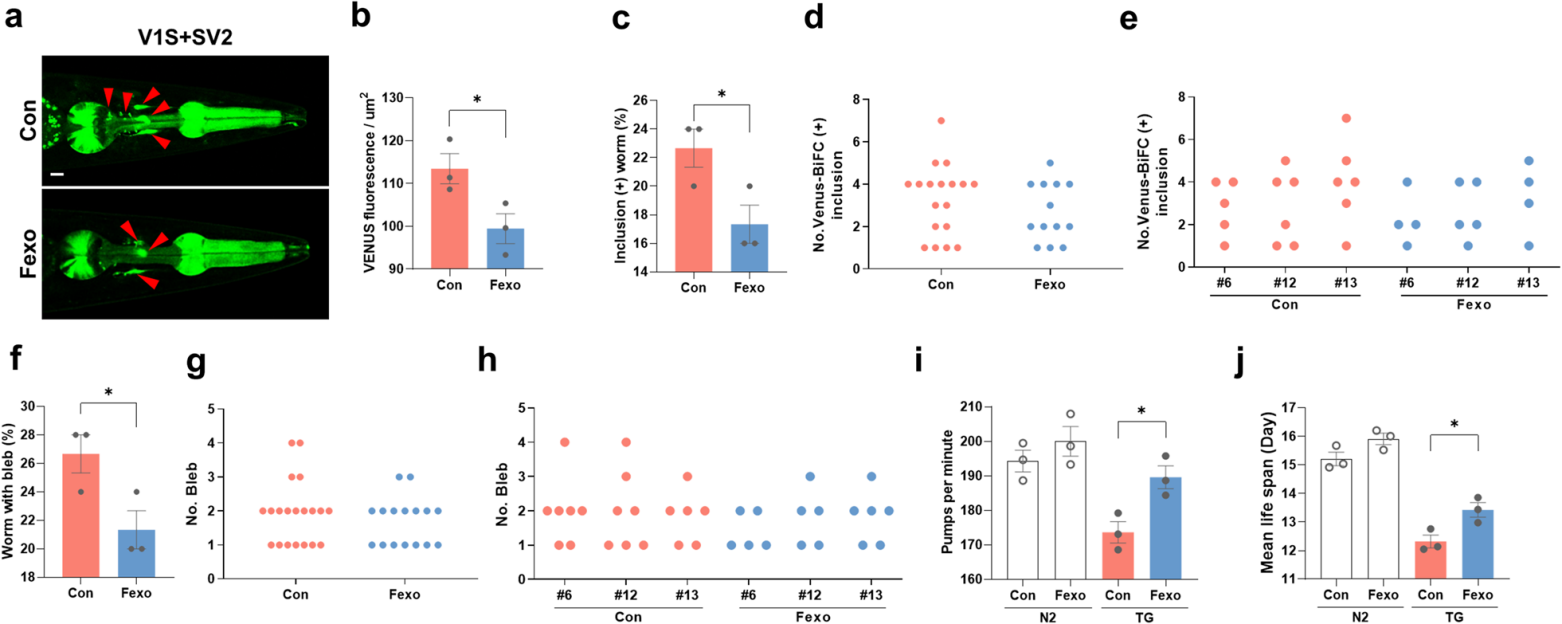
Fexofenadine alleviates the spread of α-syn pathology in *C. elegans*. **a, b** Alteration of VENUS-BiFC fluorescence in Tg models with fexofenadine at day 13. The red arrowheads point to VENUS-positive inclusions in the pharynx. Twenty-five worms for each line were used (*n* = 3 in each group). Scale bar indicates 20 μm. *p < 0.05, unpaired *t*-test. **c–e** VENUS-BiFC positive inclusions at day 13. **c** Worms with VENUS-BiFC positive inclusions were quantified at day 13. The number of inclusions in each group (**d**) and in three independent lines (**e**) are displayed as scatter plots. Twenty-five worms for each line were used (*n* = 3 in each group). *p < 0.05, unpaired *t*-test. **f–h** Change in blebbing phenotype in URA motor neurons. **f** Worms with axonal blebs were quantified. Axonal bleb numbers in respective Tg group (**g**) and in different lines (**h**) were plotted. Twenty-five worms for each line were used (*n* = 3 in each group). *p < 0.05, unpaired *t*-test. **i** Pharyngeal pumping rates with treatment of fexofenadine at day 8. Thirty worms for different line were used (*n* = 3 in each group). *p < 0.05, one-way ANOVA with Tukey’s multiple comparison test. **j** Mean lifespan analysis. One hundred worms for each line were examined (*n* = 3 in each group). *p < 0.05, one-way ANOVA with Tukey’s multiple comparison test

To further validate the effects of fexofenadine on α-syn propagation in vivo, a C. *elegans* BiFC model expressing VENUS fluorescence was used [24]. In BiFC worms treated with fexofenadine, a significant reduction in the VENUS signal level was observed on day 13 (Fig. 6a, b). Furthermore, these worms exhibited fewer VENUS-positive inclusions than the control worms (Fig. 6c). Additionally, the fexofenadine-treated models displayed fewer clumps in their BiFC signals (Fig. 6d, e). We also investigated the effect of fexofenadine on the disease phenotype in C. *elegans*. Consistent with the reduction in aggregate propagation, fexofenadine decreased the formation and number of axonal blebs (Fig. 6f–h). Fexofenadine also ameliorated the abnormal pharyngeal pumping behavior (Fig. 6i) and increased the mean lifespan of the *C. elegans* BiFC model (Fig. 6j). These results indicate that pharmacological treatment with fexofenadine delayed the progression of synucleinopathy in *C. elegans*.

### RNA-seq analysis predicts that fexofenadine reduces neuroinflammation in the PFF-injected brain

**Fig. 7:**
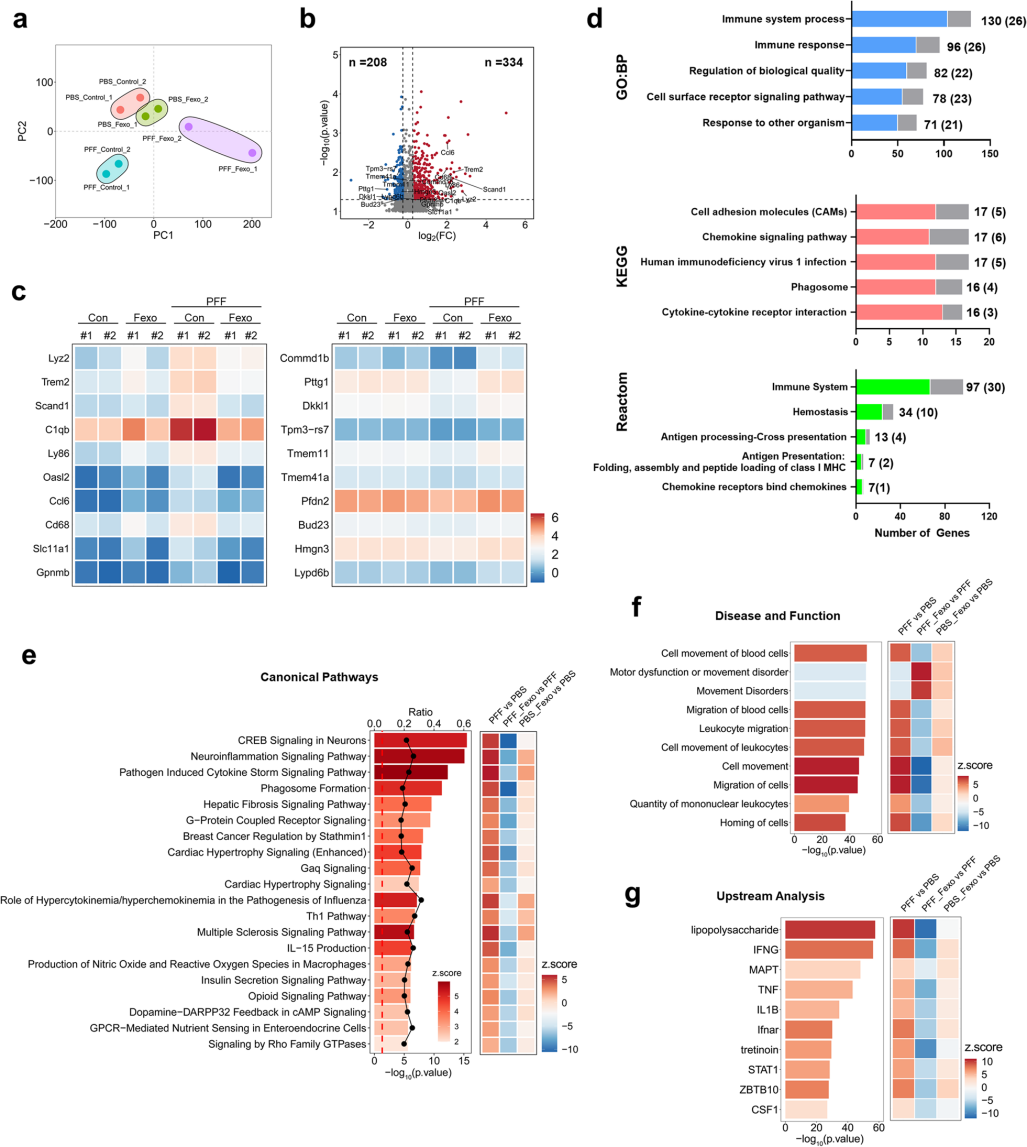
Change of gene expression profile in cortex of PFF-injected mice by fexofenadine.** a** Principal component analysis of RNA-seq data from PFF (*n* = 2), PFF + Fexo (*n* = 2), PBS (*n* = 2), and PBS + Fexo (*n* = 2) groups. **b** Volcano plot showing the number of upregulated (334) and downregulated (228) DEGs between PBS injection versus PFF injection (*x*-axis: log2(fold change); *y*-axis: − log10(*p*.value), fold change cutoff: 0.25; *p*.value cutoff < 0.05). **c** Heatmap showing top 10 genes from upregulated in PFF and downregulated in PFF + Fexo or downregulated in PFF and upregulated in PFF + Fexo. **d** GO analysis showing top 5 upregulated GO term (GO: BP, KEGG and Reactome) from DEGs of PFF versus PBS. Gray color indicates the number of genes rescued by fexofenadine. **e** Histogram showing canonical pathway by IPA of the PBS versus PFF, PFF versus PFF + Fexo, and PBS versus PBS + Fexo groups. (Bar graph: − log10 (*p*.value); line graph: ratio; color: Z-score.) **f** Histogram and heatmap showing disease and function by IPA of the PBS versus PFF, PFF versus PFF + Fexo, and PBS versus PBS + Fexo groups. **g** Histogram and heatmap showing upstream regulators by IPA of the PBS versus PFF, PFF versus PFF + Fexo, and PBS versus PBS + Fexo groups

To investigate the mechanism of fexofenadine in PFF-injected mice, RNA-seq analysis was performed on the cortex of PFF-injected mice treated with fexofenadine. Principal component analysis revealed a distinct clustering of samples from the PBS, PBS + fexofenadine, PFF, and PFF + fexofenadine groups (Fig. 7a), indicating differential gene expression patterns between the groups. DEGs analysis was conducted using two criteria: a *p*-value < 0.05 (paired *t*-test) and a fold change cutoff of 0.25. Based on these criteria, 542 genes were found to be altered in the PFF-injected mouse brain compared to the PBS-injected mouse brain. Among these genes, 334 were upregulated and 208 were downregulated (Fig. 7b). The top 10 genes upregulated in the PFF group and downregulated in the PFF + fexofenadine group, with a focus on the one exhibiting the largest fold change in expression, were *Lyz2, Trem2, Scand1, C1qb, Ly86, Oasl2, CCl6, CD68, Slc11a1,* and *Gpnmb*. Conversely, the top 10 genes downregulated in the PFF group and upregulated in the PFF + fexofenadine group were *Commd1b, Pttg1, Dkkl1, Tpm3-rs7, Tmem11, Tmem41a, Pfdn2, Bud23, Hmgn3,* and *Lypd6b* (Fig. 7c). GO analysis revealed that the upregulated genes were mainly associated with immune response or inflammation, whereas the downregulated genes were not significantly categorized. Among the genes altered by the PFF injection, 101 (90 upregulated and 11 downregulated) were rescued by fexofenadine treatment (Fig. 7d). In addition, IPA was performed using the DEGs data and the Ingenuity Pathways Knowledge Base. Canonical pathway analysis indicated that CREB signaling in neurons, the neuroinflammation signaling pathway, and the pathogen-induced cytokine storm signaling pathway were increased by PFF injection and alleviated by fexofenadine treatment (Fig. 7e). Disease and function analysis predicted a mainly association with immune cells infiltration (Fig. 7f). Upstream regulator analysis predicted IFN-γ, IL-1β, TNF-α, and IFN-α receptors as potential upstream regulators (Fig. 7g). These findings suggest that PFF injection induces inflammatory pathways, and that fexofenadine mitigates these pathways.

### Infiltration of peripheral immune cells into the brain is reduced by fexofenadine

**Fig. 8:**
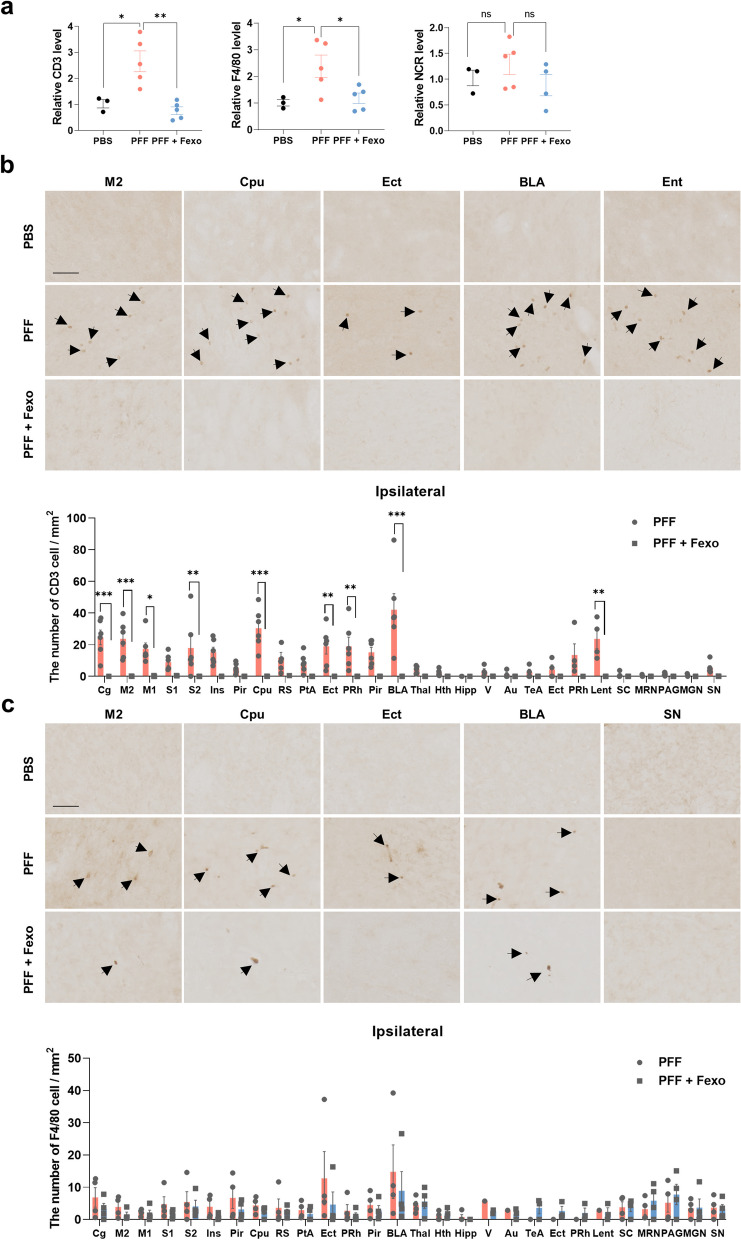
Fexofenadine alleviates the infiltration of peripheral immune cells. **a** Graph obtained from RT-PCR analysis performed on the cortex from M83 Tg mice, which were treated with PBS (*n* = 3), PFF (*n* = 5), and PFF + Fexo (*n* = 4 or 5). **p < 0.01, *p < 0.05*,* one-way ANOVA with Tukey’s multiple comparison test. **b** IHC of CD3 in the ipsilateral M2, Cpu, Ect, BLA, and Ent of M83 Tg mice brain. Values are derived from the analysis of data collected from PBS (*n* = 3), PFF (*n* = 6) or PFF + Fexo (*n* = 4) groups. ***p < 0.001, **p < 0.01, * *p* < 0.05*,* two-way ANOVA with Tukey’s multiple comparison test. **c** IHC of F4/80 in ipsilateral M2, Cpu, Ect, BLA, and SN of M83 Tg mice brain. Values are derived from the analysis of data collected from PBS (*n* = 3), PFF (*n* = 4) or PFF + Fexo (*n* = 4) groups

To validate the RNA-seq data, we performed RT-PCR for immune cell markers in brain samples. We observed an increase in the mRNA levels of the T cell marker CD3 and the macrophage marker F4/80 in the cortex 30 days after PFF injection, which were reduced by fexofenadine. There was no change in the mRNA levels of NCR1, a marker of natural killer cells (NK cells), after PFF and fexofenadine treatments (Fig. 8a). IHC analysis of CD3 showed results similar to those obtained by RT-PCR (Fig. 8b; Additional file 1: Fig. S4). F4/80 (+) cells were also increased by PFF exposure, and this increase was likely rescued by fexofenadine, although the difference was not statistically significant (Fig. 8c; Additional file 1: Fig. S5). We did not observe NCR (+) cells in any group under our experimental conditions (Additional file 1: Fig. S6), which may have been due to the infiltration of a small number of NK cells into the brain. These data suggest that fexofenadine reduces the infiltration of immune cells into the brain during α-syn propagation.

## Discussion

Evidence-based drug discovery is a widely used and valuable approach in pharmaceutical research that has yielded successful results through well-designed studies and the conduct of in vitro and in vivo experiments. We used the Korean national claims data to identify potential candidates for PD. According to our analysis, there was no difference in the LEDD between the antihistamine-exposed and unexposed groups. However, a few drugs differed when analyzed according to the individual antihistamine-exposed groups. Although there were other antihistamines with significant p-values in the t-test between the exposed and unexposed groups, we considered fexofenadine as a new drug candidate for PD because it had the most substantial effect on inhibiting the increment ratio difference between the two groups. There may be certain limitations associated with our research results. LEDD was used as an indirect surrogate measure to assess PD progression. However, it does not capture the full complexity of disease progression and may not accurately capture individual symptom variability and therapeutic responses. Additionally, the use of claims data poses a challenge in assessing medication adherence. Although claims data provide information on medication prescriptions and use, they lack concrete evidence of patients’ actual adherence to therapeutic regimens, thus limiting their confidence. Accordingly, we validated this model using various animal models of PD.

Using three different model systems, we showed that fexofenadine ameliorated PD pathology. In the 6-OHDA model, which mimics the dopaminergic neuronal cell death observed in patients with PD, fexofenadine protected dopaminergic neurons from 6-OHDA injection, as well as showing behavioral improvement. Additionally, in a PFF injection model that mimicked the spread of LBs or LNs observed in patients with PD, fexofenadine attenuated α-syn propagation. In a *C. elegans* model, fexofenadine also attenuated α-syn propagation as well as dysfunctional behaviors, suggesting that fexofenadine may affect the common targets in both 6-OHDA and α-syn propagation model systems. Neuroinflammation is a common target of these two model systems.

Numerous recent findings have suggested the involvement of a neuroinflammatory response in the neurodegeneration in idiopathic PD [26, 27]. Animal models of PD show inflammatory phenomena, such as microglial activation and cytokine production, which can modulate disease progression. In the 6-OHDA model, dopaminergic cell death always accompanies microglial activation [28, 29]. In the PFF injection model, the propagation of α-syn is also accompanied by micro/astroglial activation [18, 30, 31]. We observed that the 6-OHDA injection induced dopaminergic neuronal death accompanied by microglial activation, which was attenuated by fexofenadine. In the PFF injection model, the appearance of pSer129 α-syn-positive inclusions was accompanied by micro/astroglial activation, which was also attenuated by fexofenadine, suggesting that the main effect of fexofenadine may be related to the attenuation of neuroinflammation, which was also supported by our transcriptome analysis.

Neuroinflammation is a physiological response that protects the central nervous system (CNS) from exogenous and endogenous insults; however, uncontrolled and prolonged inflammatory responses are detrimental to the CNS. Recently, it has been demonstrated that both innate and adaptive immune cells from the periphery can penetrate the blood–brain barrier (BBB) and in the CNS, thus participating in neuroinflammation [32, 33]. Interestingly, fexofenadine is a second-generation antihistamine that does not penetrate the CNS and has the least CNS side effects among the second-generation antihistamines [34]. Accordingly, it is possible that fexofenadine affects peripheral immune cells prior to their infiltration into the brain, resulting in the attenuation of immune cell infiltration into the brain. Peripheral immune disturbances were observed after intrastriatal administration of 6-OHDA [35, 36] and PFF [37]. It has also been proposed that inflammatory mediators produced by activated microglia enter the periphery and activate peripheral immune cells [38]. Peripheral inflammation may lead to self-perpetuating neuroinflammation and progressive dopamine neurodegeneration [39], thus creating a self-perpetuating inflammatory vicious cycle [27, 40]. A growing body of evidence suggests that peripheral inflammation is associated with PD [41, 42]. Peripheral inflammation, which is reflected by increased levels of inflammatory cytokines and abnormal hyperactivation of immune cells, has been observed in patients with PD [42, 43]. Non steroidal anti-inflammatory drugs are associated with risk reduction of approximately 15% and 17% in two separate meta-analyses [44, 45], although negative results have also been published [46]. Accordingly, the peripheral effects of fexofenadine may stop the vicious inflammatory cycle and further attenuate PD pathology.

Fexofenadine has been increasingly reported to exert pleiotropic effects in addition to inhibiting histamine receptor 1. Fexofenadine significantly inhibited the release of RANTES, IL-8, GM-CSF, soluble ICAM-1, and COX-2 activity [47]. It potently inhibited NF-ĸB signaling in vitro and in vivo, and ameliorated disease symptoms [48–50], suggesting that fexofenadine has additional anti-inflammatory effects and thus prevents neuroinflammation. To date, when looking for new drugs to treat neurodegenerative diseases, the first consideration is whether or not drug enters the CNS. However, this study is significant because it demonstrates that drugs that do not enter the CNS can be effective against CNS diseases. Although we assumed that fexofenadine could not penetrate the CNS, we cannot completely rule out a direct effect of fexofenadine on the CNS. Disruption of the BBB in PD has been reported in animal models and postmortem human brains [51–53]. Therefore, fexofenadine may directly penetrate the brain. Central histaminergic systems have been reported to be altered in the brains of rats lesioned to model PD [54] and in the brains of patients with PD [55]. Pyrilamine, a first-generation antihistamine, reportedly reduces the loss of dopaminergic neurons in 6-OHDA-lesioned rats [15]. Mepyramine protects against histamine-induced dopamine neuron death in vivo [56]. To clarify the role of fexofenadine in PD more definitively, further studies are necessary.

## Conclusions

In summary, we examined the association between antihistamine use and PD progression using National Health Insurance claims data in the real world. We found that fexofenadine is a potential drug candidate for slowing PD progression. In several model systems, including 6-OHDA, PFF injection, and *C. elegans* BiFC models, we demonstrated that fexofenadine attenuated PD pathologies. RNA-seq analysis and the subsequent experiments suggested that fexofenadine blocked peripheral immune cell infiltration into the brain, which was confirmed in the PFF injection model and may attenuate PD pathology. Our innovative approach, which synergistically integrates real-world data with animal model systems, provides a promising avenue for the development of novel therapeutics targeting PD.

### Supplementary Information

**Additional file 1: Figure S1. **Schematic images of the regions annotated for pathology measures and used in subsequent analysis. Bolded regions were analyzed. Abbreviations : Cg = cingulate cortex; M1 = motor cortex 1; M2 = motor cortex 2; S1 = somatosensory cortex 1; S2 = somatosensory cortex 2; Ins = insular cortex; Pir = piriform cortex; Cpu = striatum; Sep = septal; Acb = accumbens nucleus; BST = bed nucleus of the stria terminalis; SI = substantia innominata; Hth = Hypothalamus; RS = retrosplenial cortex; PtA = parietal association cortex; Ect = ectorhinal cortex; PRh = perirhinal cortex; Ce = central amygdaloid nucleus; Me = medial amygdaloid nucelus; BLA = basolateral amygdaloid nucleus; BMA = basomedial amygdaloid nucelus; Thal = thalamic nuclei; Hipp = hippocampus; V = visual corterx; Au = auditory cortrex; TeA = temporal association cortex; Ent = entorhinal cortex; S = subiculum; SC = superior colliculus; MRN = midbrain red nucleus; MGN = medial geniculate nucleus; PAG = periaqueductal gray; VTA = ventral tegment area; SN = substantia nigra. **Figure S2. **Fexofenadine mitigates the astrogliosis in PFF-injected mice. IHC of GFAP in the ipsilateral S1, RS, Ect, BLA, Hipp, Thal, Ent, MRN, and PAG regions of PBS, PFF, and PFF + Fexofenadine (Fexo). Scale bar indicates 50 μm.** Figure S3. **Fexofenadine mitigates the microgliosis in PFF-injected mice. IHC of IBA-1 in the ipsilateral S1, RS, Ect, BLA, Hipp, Thal, Ent, MRN, and PAG regions of PBS, PFF, and PFF + Fexo. Scale bar indicates 50 μm. **Figure S4. **Fexofenadine alleviates the infiltration of CD3 positive T cells in PFF-injected mice. **a** IHC of CD3 in the ipsilateral and contralateral S2, RS, PRh, Thal, MRN, PAG, and SN regions of PBS, PFF, and PFF +Fexo. Scale bar indicates 50 μm. **b** Values are derived from the analysis of data collected from PBS (n = 3), PFF (n = 6), and PFF + Fexo (n = 4) groups. *** *p* < 0.001, ** *p* < 0.01, * *p* < 0.05, two-way ANOVA. **Figure S5. **Fexofenadine does not affect macrophages in PFF-injected mice. **a **IHC of F4/80 in the ipsilateral and contralateral S2, Pir, RS, PRh, Thal, MRN, and PAG regions of PBS, PFF, and PFF + Fexo. Scale bar indicates 50 μm. **b** Values are derived from the analysis of data collected from PBS (n = 3), PFF (n = 4), and PFF + Fexo (n = 4) groups, two-way ANOVA.** Figure S6. **NK cell does not infiltrate in PFF-injected mice. IHC of NCR1 in the ipsilateral and contralateral M2, S2, Pir, Cpu, RS, Ect, BLA, Thal, V, MRN, PAG, and SN regions of PBS (n = 3), PFF (n = 4), and PFF + Fexo (n = 4). Scale bar indicates 50 μm.** Table S1. **Real-time PCR Primers.** Table S2. **Median time (in days) differences for each antihistamine.

## Data Availability

All RNA-seq data have been submitted to the Gene Expression Omnibus (GEO) under accession number GSE241257. The datasets used and/or analyzed during the current study are available from the corresponding author on reasonable request.

## Abbreviations

PD: Parkinson’s disease
LBs: Lewy bodies
LNs: Lewy neurites
α-syn: α-Synuclein
6-OHDA: 6-Hydroxydopamine
NHIS: National Health Insurance Service
LEDD: Levodopa equivalent daily dose
LED: Levodopa equivalent dose
GFAP: Glial fibrillary acidic protein
TH: Tyrosine hydroxylase
Tg: Transgenic
Cpu: Striatum
PBS: Phosphate-buffered saline
PFF: α-Synuclein preformed fibrils
SN: Substantia nigra
BiFC: Bimolecular fluorescence complementation
NGM: Nematode growth medium
GO: Gene Ontology
IPA: Ingenuity pathways analysis
DEGs: Differentially expressed genes
RT-PCR: Real-time PCR
NK cells: Natural killer cells
CNS: Central nervous system
BBB: Blood–brain barrier
