# Supplementary material for: Integration of National Health Insurance claims data and animal models reveals fexofenadine as a promising repurposed drug for Parkinson’s disease
Source: J Neuroinflammation. 2024 Feb 21;21:53. doi: 10.1186/s12974-024-03041-7 (PMC10880337; doi:10.1186/s12974-024-03041-7)
Supplement: Supplementary file 1 — Additional file 1: Figure S1. Schematic images of the regions annotated for pathology measures and used in subsequent analysis. Bolded regions were analyzed. Abbreviations : Cg = cingulate cortex; M1 = motor cortex 1; M2 = motor cortex 2; S1 = somatosensory cortex 1; S2 = somatosensory cortex 2; Ins = insular cortex; Pir = piriform cortex; Cpu = striatum; Sep = septal; Acb = accumbens nucleus; BST = bed nucleus of the stria terminalis; SI = substantia innominata; Hth = Hypothalamus; RS = retrosplenial cortex; PtA = parietal association cortex; Ect = ectorhinal cortex; PRh = perirhinal cortex; Ce = central amygdaloid nucleus; Me = medial amygdaloid nucelus; BLA = basolateral amygdaloid nucleus; BMA = basomedial amygdaloid nucelus; Thal = thalamic nuclei; Hipp = hippocampus; V = visual corterx; Au = auditory cortrex; TeA = temporal association cortex; Ent = entorhinal cortex; S = subiculum; SC = superior colliculus; MRN = midbrain red nucleus; MGN = medial geniculate nucleus; PAG = periaqueductal gray; VTA = ventral tegment area; SN = substantia nigra. Figure S2. Fexofenadine mitigates the astrogliosis in PFF-injected mice. IHC of GFAP in the ipsilateral S1, RS, Ect, BLA, Hipp, Thal, Ent, MRN, and PAG regions of PBS, PFF, and PFF + Fexofenadine (Fexo). Scale bar indicates 50 μm. Figure S3. Fexofenadine mitigates the microgliosis in PFF-injected mice. IHC of IBA-1 in the ipsilateral S1, RS, Ect, BLA, Hipp, Thal, Ent, MRN, and PAG regions of PBS, PFF, and PFF + Fexo. Scale bar indicates 50 μm. Figure S4. Fexofenadine alleviates the infiltration of CD3 positive T cells in PFF-injected mice. a IHC of CD3 in the ipsilateral and contralateral S2, RS, PRh, Thal, MRN, PAG, and SN regions of PBS, PFF, and PFF +Fexo. Scale bar indicates 50 μm. b Values are derived from the analysis of data collected from PBS (n = 3), PFF (n = 6), and PFF + Fexo (n = 4) groups. *** p < 0.001, ** p < 0.01, * p < 0.05, two-way ANOVA. Figure S5. Fexofenadine does not affect macrophages in PFF-injec [file 12974_2024_3041_MOESM1_ESM.pdf]

Additional file 1

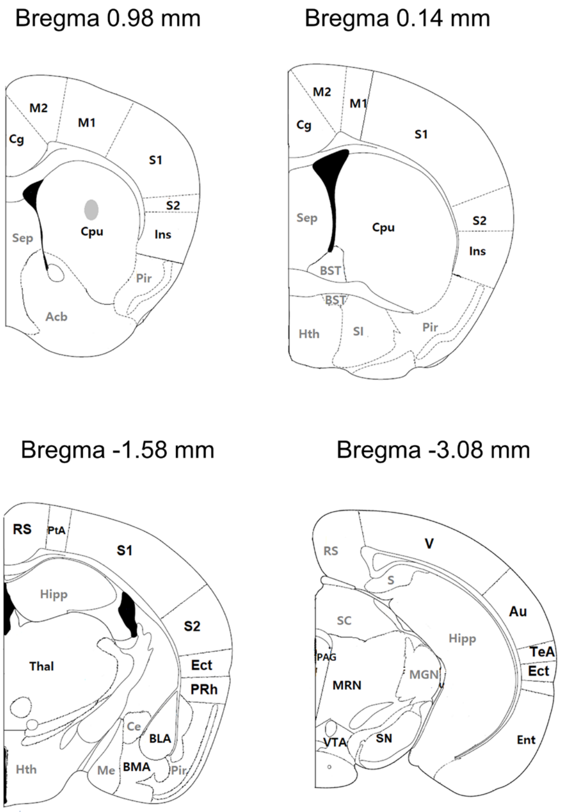

Fig. S1 Schematic images of the regions annotated for pathology measures and used in subsequent analysis. Bolded regions were analyzed. Abbreviations : Cg = cingulate cortex; M1 = motor cortex 1; M2 = motor cortex 2; S1 = somatosensory cortex 1; S2 = somatosensory cortex 2; Ins = insular cortex; Pir = piriform cortex; Cpu = striatum; Sep = septal; Acb = accumbens nucleus; BST = bed nucleus of the stria terminalis; SI = substantia innominata; Hth = Hypothalamus; RS = retrosplenial cortex; PtA = parietal association cortex; Ect = ectorhinal cortex; PRh = perirhinal cortex; Ce = central amygdaloid nucleus; Me = medial amygdaloid nucelus; BLA = basolateral amygdaloid nucleus; BMA = basomedial amygdaloid nucelus; Thal = thalamic nuclei; Hipp = hippocampus; V = visual corterx; Au = auditory cortrex; TeA = temporal association cortex; Ent = entorhinal cortex; S = subiculum; SC = superior colliculus; MRN = midbrain red nucleus; MGN = medial geniculate nucleus; PAG = periaqueductal gray; VTA = ventral tegment area; SN = substantia nigra

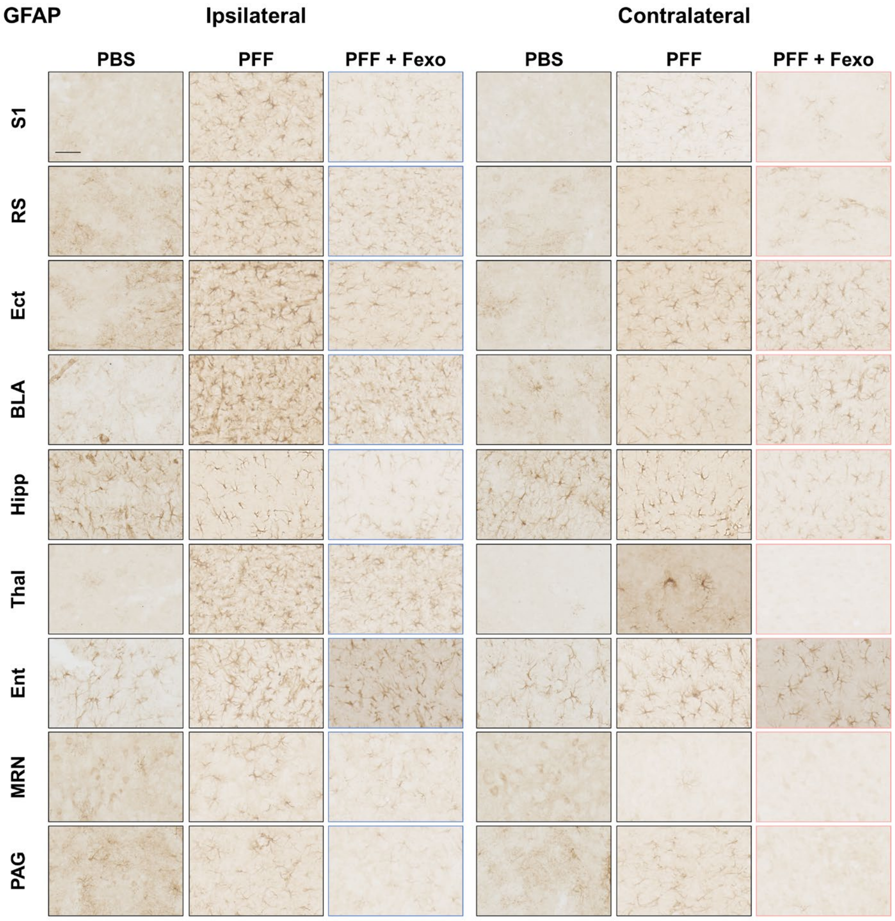

Fig. S2 Fexofenadine mitigates the astrogliosis in PFF-injected mice. IHC of GFAP in the ipsilateral S1, RS, Ect, BLA, Hipp, Thal, Ent, MRN, and PAG regions of PBS, PFF, and PFF + Fexofenadine (Fexo). Scale bar indicates 50 μm.

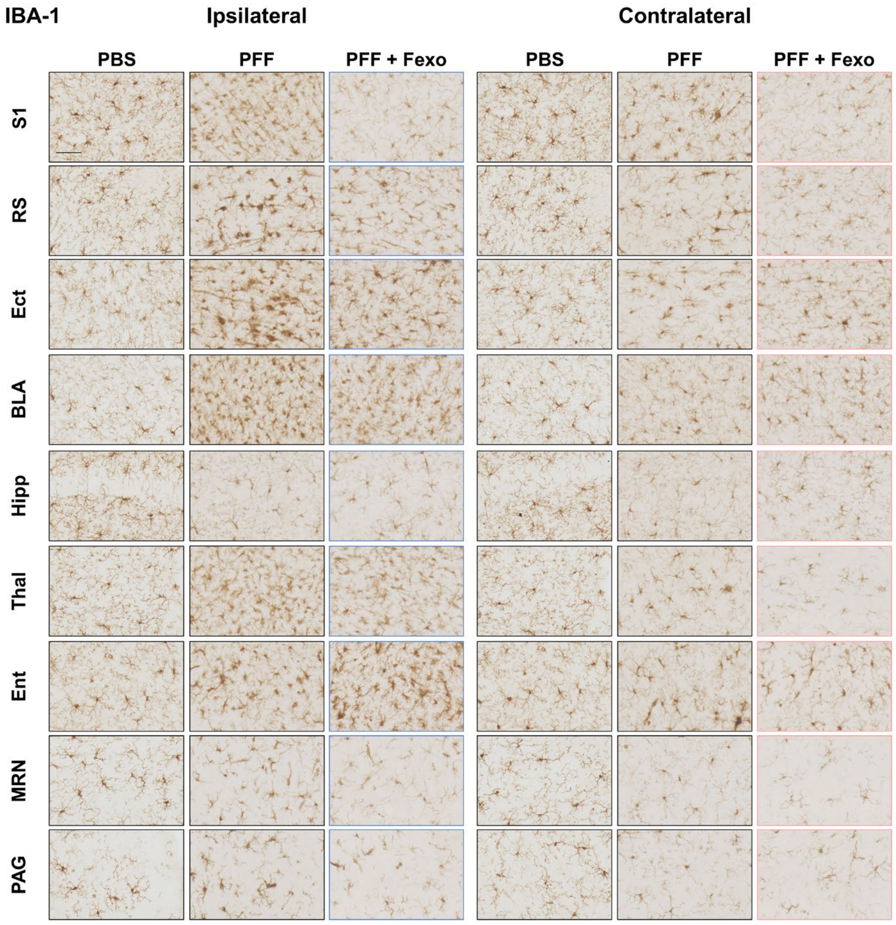

Fig. S3 Fexofenadine mitigates the microgliosis in PFF-injected mice. IHC of IBA-1 in the ipsilateral S1, RS, Ect, BLA, Hipp, Thal, Ent, MRN, and PAG regions of PBS, PFF, and PFF + Fexo. Scale bar indicates 50 μm

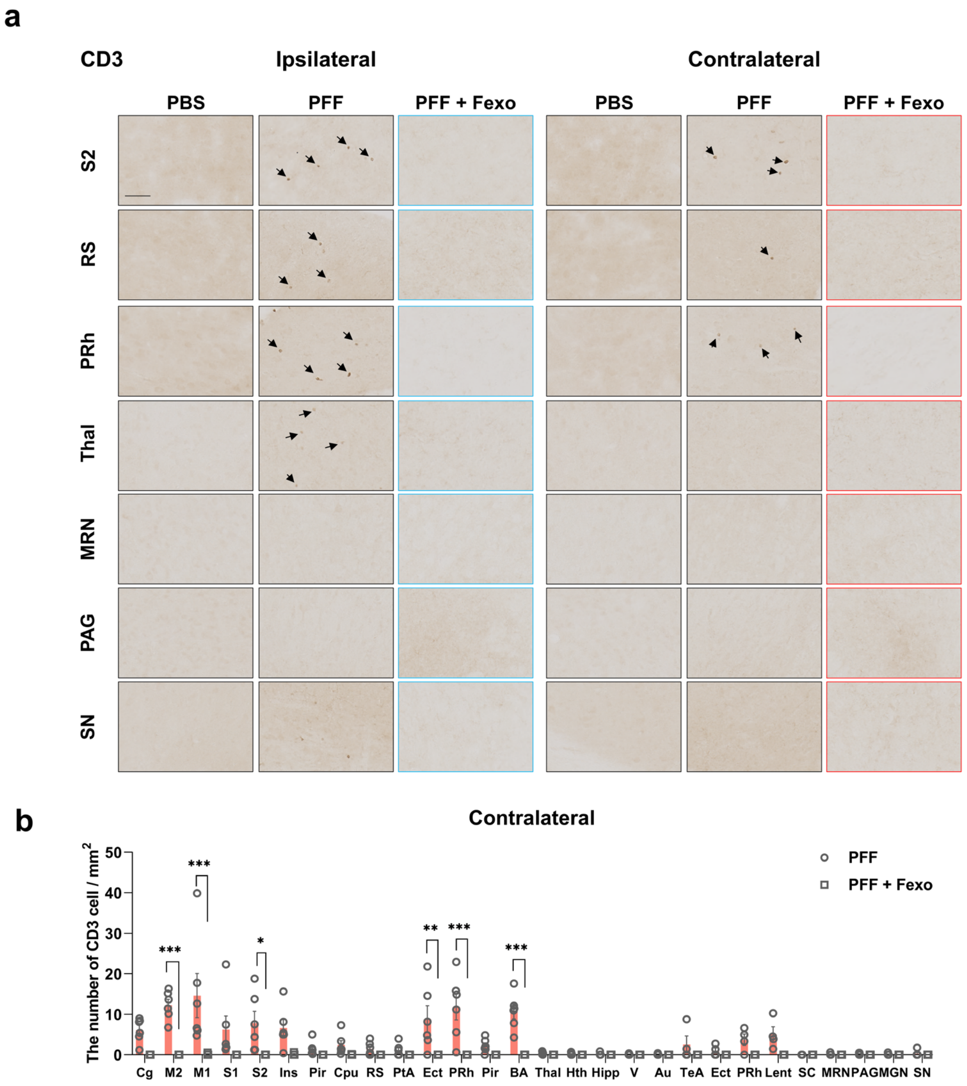

Fig. S4 Fexofenadine alleviates the infiltration of CD3 positive T cells in PFF-injected mice. a IHC of CD3 in the ipsilateral and contralateral S2, RS, PRh, Thal, MRN, PAG, and SN regions of PBS, PFF, and PFF +Fexo. Scale bar indicates 50 µm. **b** Values are derived from the analysis of data collected from PBS (n = 3), PFF (n = 6), and PFF + Fexo (n = 4) groups. *** $p < 0.001$, ** $p < 0.01$, * $p < 0.05$, two-way ANOVA.

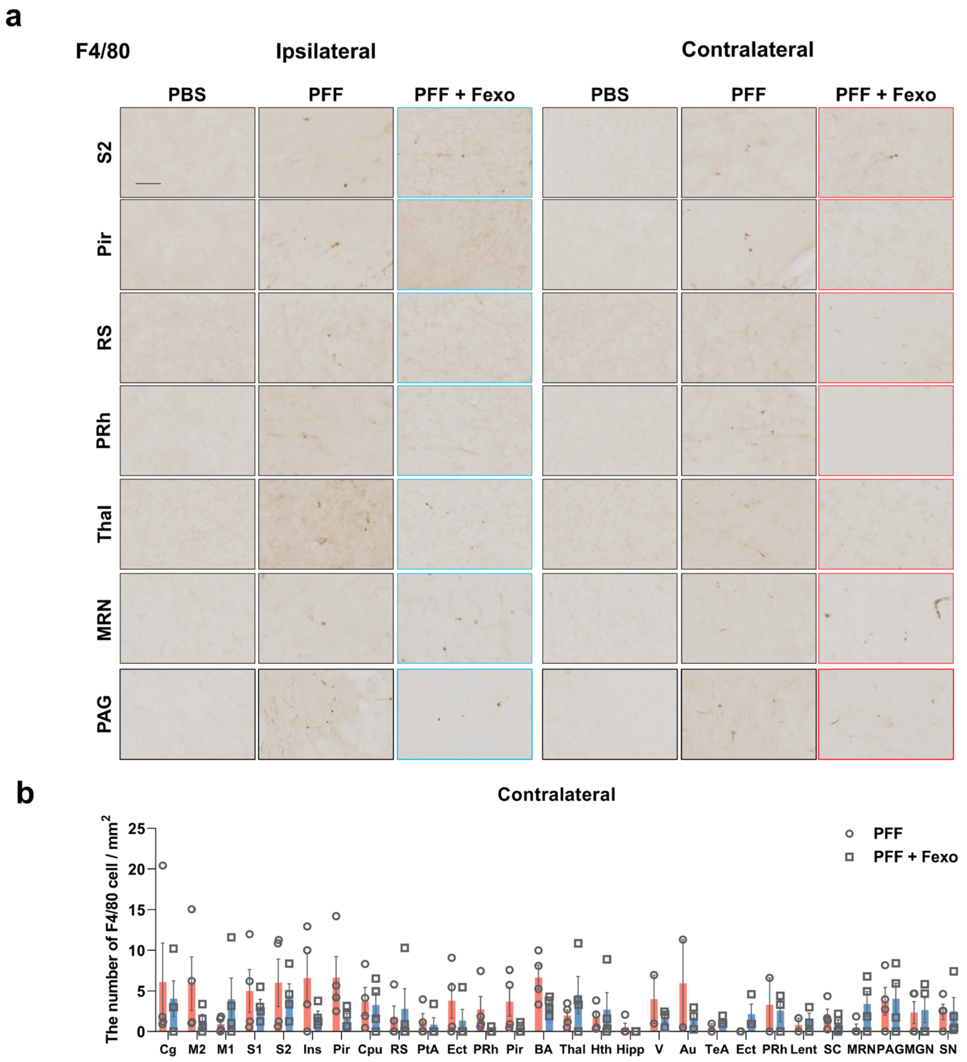

Fig. S5 Fexofenadine does not affect macrophages in PFF-injected mice. a IHC of F4/80 in the ipsilateral and contralateral S2, Pir, RS, PRh, Thal, MRN, and PAG regions of PBS, PFF, and PFF + Fexo. Scale bar indicates 50 μm. **b** Values are derived from the analysis of data collected from PBS (n = 3), PFF (n = 4), and PFF + Fexo (n = 4) groups, two-way ANOVA.

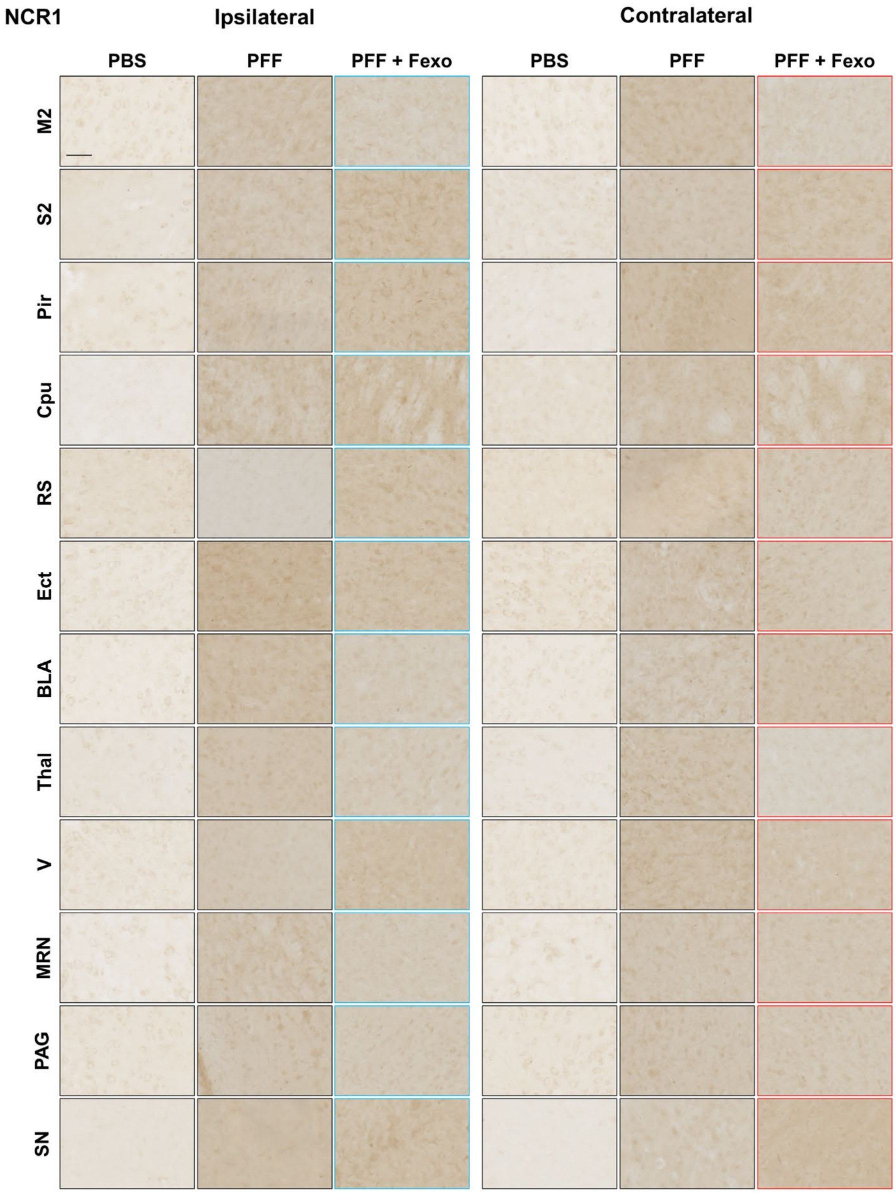

Fig. S6 NK cell does not infiltrate in PFF-injected mice. IHC of NCR1 in the ipsilateral and contralateral M2, S2, Pir, Cpu, RS, Ect, BLA, Thal, V, MRN, PAG, and SN regions of PBS (n = 3), PFF (n = 4), and PFF + Fexo (n = 4). Scale bar indicates 50 μm.

Table. S1 Real-time PCR Primers.

Gene	Forward Primer	Reverse Primer
CD3	5'-TGGAGAAGCAAAGAGACTGACA-3'	5'-GCCATCCACTTGTACCAAATTC-3'
F4/80	5'-TTTCCTCGCCTGCTTCTTC-3'	5'-CCCCGTCTCTGTATTCAACC-3'
NCR1	5'-GAGCCAGAGGATCAACACTGAAA-3'	5'-GGTAACATTCTCCCCCAAGGT-3'
GAPDH	5'-TGCACCACCAACTGCTTAG-3'	5'-GGATGCAGGGATGATGTTC-3'

Table. S2 Median time (in days) differences for each antihistamine.

Antihistamine	Median time (days)
**Overall**	752
**1st Genetrarion**	766
Chlorpheniramine	1022
Clemastine	490
Dimenhydrinate	515
Hydroxyzine	723
**2nd Genetration**	723
Azelastine	662
Bepotastine	793
Cetirizine	638
Desloratadine	1006
Ebastine	670
Fexofenadine	703
Ketotifen	381
Levocetirizine	777
Loratadine	484
Olopatadine	699
